# *CYP2C19* polymorphism affects single-dose pharmacokinetics of oral pantoprazole in healthy volunteers

**DOI:** 10.1007/s00228-012-1252-3

**Published:** 2012-03-15

**Authors:** Barbara Gawrońska-Szklarz, Urszula Adamiak-Giera, Elżbieta Wyska, Mateusz Kurzawski, Wanda Gornik, Maria Kaldonska, Marek Drozdzik

**Affiliations:** 1 Department of Pharmacology, Pomeranian Medical University, Powstancow Wlkp 72, 70-111 Szczecin, Poland; 2 Department of Pharmacokinetics and Physical Pharmacy, Jagiellonian University, Collegium Medicum, Cracow, Poland

**Keywords:** *CYP2C19* polymorphism, Pantoprazole, Pharmacokinetics

## Abstract

**Objectives:**

Pantoprazole is metabolized by cytochrome P450 2 C19, which shows genetic polymorphism. The effect of *CYP2C19* polymorphism on single-dose pharmacokinetics of oral pantoprazole in healthy volunteers was evaluated.

**Methods:**

Pantoprazole pharmacokinetics was determined in 32 healthy volunteers after a 40-mg single oral dose of the drug.

**Results:**

Carriers of CYP2C19*2/*2 (*n* = 2) were characterized by higher, starting from 3.5 h post dose, plasma concentrations of pantoprazole in comparison to wild-type (CYP2C19*1/*1, *n* = 6) volunteers. In subjects with CYP2C19*17/*17 genotype (*n* = 6) significantly lower plasma concentrations of the drug vs CYP2C19*1/*1 carriers, were observed from 3.0 h after oral pantoprazole administration. Carriers of CYP2C19*1/*17 (*n* = 6) and CYP2C19*2/*17 (*n* = 6) displayed concentration–time profiles comparable to wild-type subjects. CYP2C19*2/*2 volunteers showed a decrease in terminal elimination rate constant (λ_z_) by 83.3%, prolongation of terminal half-life (t_½_) by 572%, a rise in area under the concentration–time curve (AUC) and mean residence time (MRT) by 506% and 259% respectively. Heterozygotes, i.e.. CYP2C19*1/*2 vs CYP2C19*1/*1 were characterized by higher AUC (4.38 ± 1.00 mg⋅h/L vs 3.00 ± 1.02 mg⋅h/L, *p* < 0.05) and C_max_ (2.13 ± 0.42 mg/L vs 1.61 ± 0.35 mg/L, *p* < 0.05) respectively. A significant reduction in MRT (3.83 ± 0.82 h vs 2.73 ± 0.23 h, *p* < 0.05) in carriers of CYP2C19*17/*17 vs CYP2C19*1/*1 genotypes was observed. Population modeling confirmed the influence of *1/*2, *2/*2, and *17/*17 genotypes on the pharmacokinetics of pantoprazole. The lowest population oral clearance was assessed in the carriers of genotype *2/*2 (3.68 L/h) and the highest value in subjects with genotype *17/*17 (31.13 L/h).

**Conclusion:**

These data suggest that *CYP2C19* polymorphism is an important determinant of pantoprazole pharmacokinetics.

## Introduction

CYP2C19 is an important drug-metabolizing enzyme encoded by a highly polymorphic gene, with the two most frequent allelic variants in Caucasian populations, CYP2C19*2 and CYP2C19*17. Population studies have demonstrated that subjects homozygous for the inactivating allele CYP2C19*2 associated with a splicing defect are phenotypically poor metabolizers of CYP2C19 substrates [1, 2]. Poor metabolizers are found in 3–5% of Caucasians, and much more frequently in Asian populations, i.e., 12–23% [3]. The CYP2C19*17 allele was documented to correlate with high CYP2C19 activity [4]. In European populations the CYP2C19*17 allele is found in 18–28% of subjects, in 17–18% of Africans and 0.3–4% in Asian populations [5].

Proton pump inhibitors (PPI), among them pantoprazole, are the mainstays in the treatment of gastric acid-related disorders. It was documented that the pharmacokinetics of PPI as well as the clinical outcome of treatment are associated with *CYP2C19* polymorphism, being their main metabolizing enzyme. However, the effect of *CYP2C19* on pantoprazole metabolism was not as extensively studied as in the case of omeprazole. Tanaka et al. reported that in poor *CYP2C19* metabolizers metabolism of R(+)-pantoprazole was impaired to a greater extent than that of S(-)-pantoprazole [6]. Some studies on the effects of the *CYP2C19* polymorphism on pantoprazole kinetics revealed an association of drug pharmacokinetics and genotypes in adults and children [7–9]. However, these studies were carried out on a limited number of cases and did not involve CYP2C19*17/*17 homozygotes, which seems to be an important determinant in pantoprazole metabolism.

Our previous preliminary study also suggested an impact of the *CYP2C19* polymorphism on pantoprazole metabolism. It was found that concentrations of the drug at 3 h post-oral dose were the highest in heterozygous subjects, carriers of CYP2C19*1/*2 genotypes (unfortunately, we were not able to measure pantoprazole concentrations in CYP2C19*2/*2 patients), and the lowest in the case of CYP2C19*17/*17 subjects [10]. Therefore, it was decided to study an association between *CYP2C19* polymorphism and single-dose pharmacokinetics of oral pantoprazole in healthy volunteers.

## Materials and methods

### Subjects

In the first part of the study 120 healthy, unrelated volunteers of Caucasian origin, Polish nationality (52 males and 68 females; age rank 20–27 years) were genotyped for *CYP2C19* polymorphism. Informed consent was obtained from all participants. The study was approved by the Ethics Committee of Pomeranian Medical University, Szczecin, Poland. Within the genotyped group, 46 subjects were homozygous: 38 for the CYP2C19*1 allele, 6 for the CYP2C19*17 allele, and 2 for the CYP2C19*2 allele. Seventy-four were heterozygous with the following *CYP2C19* genotypes: 20 participants with *1/*2, 38 with *1/*17, and 16 with *2/*17 genotypes.

In the second part of the study pharmacokinetics of pantoprazole after a single oral dose of 40 mg in 32 healthy volunteers defined for specific *CYP2C19* genotypes was determined. Subject characteristics and genotypes are shown in Table 1.

**Table 1:** Subject characteristics and *CYP2C19* genotypes (*n* = 32)

Parameter	*1/*1	*1/*2	1/*17	*2/*2	*2/*17	*17/*17
Gender (male/female)	6 (3/3)	6 (3/3)	6 (3/3)	2 (2/0)	6 (3/3)	6 (3/3)
Age (years) mean (range)	23 (21–26)	22 (20–24)	23 (22–24)	23 (22,24)	24 (23–26)	24 (23–25)
Weight (kg) mean (range)	75 (56–90)	66 (53–78)	69 (56–83)	88 (80–96)	66 (52–76)	68 (53–81)

### Genotyping

*CYP2C19* genotyping was performed in all study subjects. Genomic DNA was extracted from 200 μL of whole blood samples using GeneMATRIX Quick Blood DNA Purification Kit (EURx, Poland). Each individual was genotyped for a presence of SNPs marking *CYP2C19* variant alleles: rs4244285 (681 G > A) for allele *2, and rs12248560 (−806 C > T) for allele *17. The allelic discrimination TaqMan real-time PCR assays (Assay IDs: C_25986767_70, C_469857_10, Applied Biosystems, USA) were used for detection. Fluorescence data were captured using an ABI PRISM 7500 FAST Real-Time PCR System (Applied Biosystems), after 40 cycles of PCR.

### Drug concentration analysis

Pantoprazole concentration in plasma was determined by a validated high-performance liquid chromatography method (HPLC) with ultraviolet detection following solid phase extraction [11]. The chromatographic system consisted of a pump HP 1100, autosampler HP 1100, an ultraviolet detector HP 1100 and DAD detector Agilent 1100. The isocratic mobile phase was composed of a buffer solution consisting of 25 mM potassium phosphate monobasic in water containing 0.25% triethylamine and acetonitrile (35:65% v/v). The pH = 6.5 was adjusted with phosphoric acid. The mobile phase was pumped at an isocratic flow rate of 1 mL/min at a temperature of 25°C. The wavelength of UV detection was set at 290 nm. Chromatographic separations were achieved on Supelcosil LC-8-DB column (5 μm, 15 × 4.6 mm). Solid-phase extraction was used to extract the pantoprazole and tinidazole as an internal standard. Recoveries from human plasma were ≥ 90% for pantoprazole. The lower limit of detection (LOD) at a signal:noise ratio 3:1 was 0.012 mg/L and the lower limit of quantitation for pantoprazole was 0.025 mg/L. Intra- and interassay coefficients of variation were consistently ≤10% for plasma pantoprazole concentrations within the range of linearity. No interfering peaks from metabolites of pantoprazole and endogenous compounds were observed at the retention time of analytes. All study samples were analyzed within 3 months of their collection.

### Pharmacokinetic study

Pharmacokinetic parameters of pantoprazole such as clearance/F (CL/F, where F is the systemically available fraction of a dose), volume of distribution (Vz/F), terminal half-life (t_½_), mean residence time (MRT), time to maximum concentration (t_max_), and the maximum concentration (C_max_) were derived by noncompartmental analysis using Phoenix WinNonlin 6.2 (Pharsight Corporation, Mountain View, CA, USA). For each individual the terminal elimination rate constant (λ_z_) was determined by log-linear regression of the terminal phase of the plasma concentration–time curve. The area under the concentration–time curve (AUC) was determined by the linear trapezoidal rule from time zero to the time of the last observed concentration [12].

Differences between genotypes were evaluated using *t* test for independent samples. The level of significance was set at 0.05.

### Population pharmacokinetic modeling

Because it was impossible to assess statistically the influence of rare genotypes, e.g., CYP2C19*2/*2 on pharmacokinetics of pantoprazole using standard statistical tests, an attempt was made to evaluate the impact of CYP2C19*2 and CYP2C19*17 genotypes when included in the population pharmacokinetic model as covariates.

To assess the influence of *CYP2C19* genotypes on pantoprazole pharmacokinetics, drug concentrations were analyzed using the nonlinear mixed effects modeling program, NONMEM (version V, level 1.1). The one- and two-compartment models with first-order absorption from the central compartment and a lag time were tested. These models were implemented in the PREDPP library subroutine ADVAN 2 TRANS 2 or ADVAN4 TRANS4 in NONMEM respectively. Pharmacokinetic analysis was performed using the first-order (FO) estimation method with post-hoc estimation of individual parameters.

The intersubject variability in pharmacokinetic parameters was estimated using an exponential model:

$$ {{\text{P}}_{{ij}}} = TVP \bullet \exp \left( {{\eta_{{ij}}}} \right) $$

where P_i_ is the jth pharmacokinetic parameter for the ith subject, TVP is the typical value of jth parameter, η_ij_ is a random variable for the ith individual in the jth parameter. It is assumed that the values of *η*_*i*_ are normally distributed with a mean of zero and a variance of ω^2^.

Residual variability was described by a combined proportional and additive error model:

$$ {C_{{OBS}}} = {C_{{PRED}}}\left( {1 + {\varepsilon_1}} \right) + {\varepsilon_2} $$

where C_OBS_ and C_PRED_ are observed and predicted pantoprazole concentrations, and ε_1_ and ε_2_ are the residual intrasubject variability with means of zero and variances of σ_1_^2^ and σ_2_^2^ respectively.

The possible relationship between the typical value of the oral clearance (CL/*F*) and *CYP2C19* genetic polymorphisms was modeled according to the following equation:

$$ TVCL/F = {q_1}x{q_2}^{{G1}}x\quad .....{ }x{q_6}^{{G5}} $$

where *θ*_1_ is the population value of oral clearance for the wild-type group, *θ*_*2 -*_ *θ*_*6*_ are the fractional changes in the oral clearance for different genotypes, and G_1_ – G_5_ represent *CYP2C19* genotypes: *1/*2, *1/*17, *2/*17, *2/*2, and*17/*17 respectively.

Model building was performed using a stepwise approach, adding an additional covariate at each step. The effect of each covariate was evaluated based on changes in the objective function value (OFV). The results were considered statistically significant if the decreases in OFV were 3.84 units (*p* < 0.05), 6.63 units (*p* < 0.01), and 10.83 units (*p* < 0.001). To assess the significance of each covariate a backward elimination procedure was used. An increase in OFV greater than 6.63 (*P* < 0.01) was required to retain the covariate in the final model.

The goodness-of-fit of each analysis was assessed by the percentage relative standard error (%RSE) of the mean of parameters estimates, changes in the estimates of intersubject and residual variability, and visual inspection of scatterplots representing population (PRED) and individual (IPRED) predicted concentrations versus observed concentrations and weighted residuals (WRES) versus predicted pantoprazole concentrations. In addition, epsilon shrinkage defined as 1-SD(IWRES) was calculated in order to ensure validity of the diagnostics. Graphical diagnostics were obtained using Visual-NM, v. V [13].

## Results

**Fig. 1:**
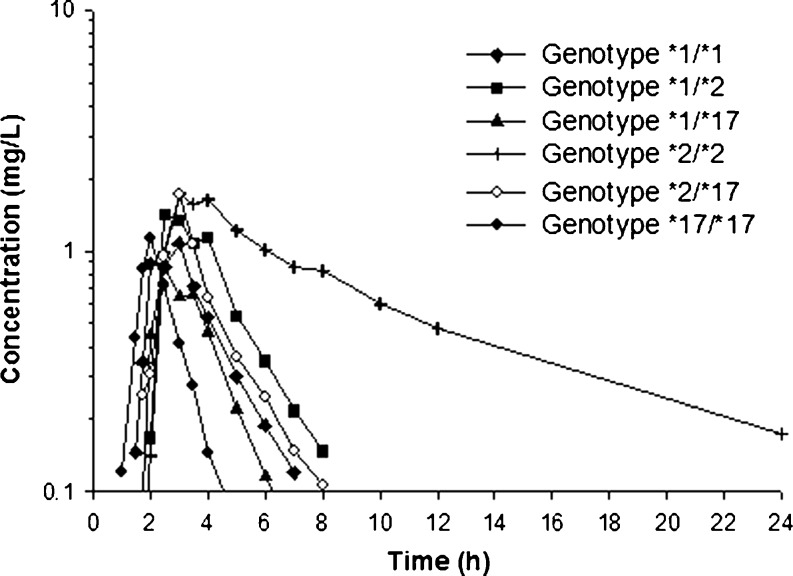
Mean plasma concentration–time profiles in subjects with different *CYP2C19* genotypes after administration of 40 mg pantoprazole as a single oral dose. Concentrations of CYP2C19*17/* were significantly different from CYP2C19*1/*1 at each time point starting from 3.0 h post-dose. Number of subjects per group *n* = 6, except for CYP2C19*2/*2 where *n* = 2

Plasma concentration versus time profiles of pantoprazole in healthy volunteers stratified according to the *CYP2C19* genotypes are presented in Fig. 1. Carriers of CYP2C19*2/*2 (*n* = 2) were characterized by considerably higher, starting from 3.5 h post dose, plasma concentrations of pantoprazole in comparison to wild-type (CYP2C19*1/*1; *n* = 6) volunteers. In subjects with the CYP2C19*17/*17 genotype (*n* = 6) significantly lower plasma concentrations of the drug vs CYP2C19*1/*1 (*n* = 6) carriers, were observed from 3.0 h after oral pantoprazole administration. Carriers of CYP2C19*1/*17 (*n* = 6) and CYP2C19*2/*17 (*n* = 6) displayed concentration–time curves comparable to wild-type subjects.

Changes in pantoprazole pharmacokinetics in subjects with different *CYP2C19* genotypes are also reflected in altered pharmacokinetic parameters (Table 2). As in the case of the drug concentrations, most prominent deviations from wild-type genotype carriers were observed in CYP2C19*2/*2 (*n* = 2) and CYP2C19*17/*17 (*n* = 6) subjects. In CYP2C19*2/*2 (*n* = 2) volunteers a marked decrease in λ_z_ by 83.3%, prolongation of t_½_ by 572% and MRT by 259%, a rise of AUC by 506% and C_max_ by 32.3% respectively were noted. In carriers of CYP2C19*17/*17 (*n* = 6) significant reduction in MRT by 24.8% (*p* < 0.05) was revealed. Subjects with CYP2C19*1/*2 (*n* = 6) genotype were characterized by markedly higher C_max_ by 32.3% (*p* < 0.05) and a significant increase in AUC by 146% (*p* < 0.05).

**Table 2:** Pharmacokinetic parameters of pantoprazole (mean ± SD) in healthy volunteers according to the *CYP2C19* genotypes

Parameter/genotype	*1/*1 (*n* = 6)	*1/*2 (*n* = 6)	*1/*17 (*n* = 6)	*2/*2 (*n* = 2)	*2/*17 (*n* = 6)	*17/*17 (*n* = 6)
λ_z_ , h^−1^	0.60 ± 0.22	0.43 ± 0.11	0.71 ± 0.14	0.10 ± 0.01	0.46 ± 0.06	0.79 ± 0.36
		[−0.354, 0.005]^a^	[−0.357, 0.565]	[−0.669, −0.347]	[−0.549, 0.260]	[−0.125, 0.497]
t_½,_ h	1.27 ± 0.41	1.69 ± 0.37	1.01 ± 0.17	7.27 ± 0.92	1.53 ± 0.20	0.99 ± 0.29
		[0.016, 0.823]	[−0.846, 0.320]	[5.052, 6.945]	[−0.338, 0.857]	[−0.653, 0.082]
t_max,_ h	2.42 ± 0.49	3.00 ± 0.63	2.83 ± 0.75	3.25 ± 1.06	2.75 ± 0.42	1.96 ± 0.33
		[−0.002, 1.168]	[−0.436, 1.269]	[−0.266, 1.933]	[−0.396, 1.063]	[−0.892, −0.025]
C_max,_ mg/L	1.61 ± 0.35	2.13 ± 0.43*	1.30 ± 0.29	1.91 ± 0.07	1.86 ± 0.55	1.30 ± 0.55
		[0.116, 0.931]	[−0.919, 0.303]	[0.036, 0.547]	[−0.476, 0.974]	[−0.780, 0.173]
AUC, mg⋅h/L	3.00 ± 1.02	4.38 ± 1.00*	2.13 ± 0.50	15.18 ± 0.07	3.56 ± 0.65	1.87 ± 0.72
		[0.333, 2.418]	[−1.817, 0.067]	[11.426,12.921]	[−0.429, 1.548]	[−2.043, −0.216]
V_z_/F, L	25.03 ± 4.63	22.92 ± 6.14	28.48 ± 8.16	27.65 ± 3.61	25.39 ± 5.42	31.88 ± 9.23
		[−7.721, 3.512]	[0.715, 6.184]	[−2.269, 7.513]	[−2.060, 2.788]	[−0.687, 14.393]
CL/F, L/h	15.22 ± 7.13	9.55 ± 2.20	19.71 ± 4.75	2.64 ± 0.01	11.55 ± 2.14	24.41 ± 9.71
		[−11.125, −0.229]	[1.854, 7.123]	[−17.795, −7.382]	[−6.006, −1.351]	[0.382, 17.981]
MRT, h	3.39 ± 0.55	4.03 ± 0.44	3.53 ± 0.68	8.78 ± 0.52	3.78 ± 0.34	2.55 ± 0.19*
		[0.062, 1.412]	[−0.998, 0.952]	[6.498, 9.716]	[−0.492, 1.234]	[−1.723, −0.477]

AUC, area under the (receiver operating characteristic) curve; V_z_ , volume of distribution ; CL/F, clearance/sysystematically available fraction of a dose; MRT, mean residence time (MRT)

^a^ 95% confidence interval (CI) on the difference between means

*p < 0.05, Student’s *t* test, vs genotype *1/*1

Population modeling confirmed a strong influence of *1/*2, *2/*2 and *17/*17 genotypes on the pharmacokinetics of pantoprazole. Based on the goodness-of-fit criteria, serum pantoprazole concentration versus time profiles were best described by a two-compartment model with first-order absorption from the central compartment and a lag time. Following preliminary analyses, the random effects were included on oral clearance (CL/F), volume of the central compartment (V_c_/F), first order absorption rate constant (k_a_), and the absorption lag time (t_lag_). Volume of peripheral compartment (V_p_/F) and intercompartmental clearance (Q/F) were the remaining model parameters. As shown in Table 3, genetic polymorphism of *CYP2C19* significantly affected pantoprazole oral clearance. Based on the changes in OFV, the strongest impact on this parameter was observed in subjects with genotype *1/*2 and in both homozygotes *2/*2, *17/*17, whereas in heterozygotes CYP2C19*17 the effect was significant but less pronounced. In addition, their inclusion in the model did not lead to a decrease in interindividual variability in CL/F. When backward deletion was performed, for genotypes *1/*17 and *2/*17 differences in OFV were less than 6.63 and, in consequence, they were removed from the model (Table 3). The estimates of final population model parameters are presented in Table 4. When comparing the basic and the final model, the interindividual variability in CL was reduced from 92.95% to 35.92% after adding CYP2C19*1/*2, *2/*2, and *17/*17 genotypes as model covariates. As expected, the lowest population oral clearance was assessed in the carriers of genotype 2*/2* (3.68 L/h) and the highest value in subjects with genotype *17/*17 (31.13 L/h). The value of this parameter for *1/*2 genotype was 8 L/h. None of other covariates, such as age, gender, and body weight significantly influenced any of the pharmacokinetic parameters of pantoprazole (data not shown).

**Table 3:** Summary of the covariate model-building steps (G1-*1/*2, G2-*1/*17, G3-*2/*17, G4-*2/*2, G5-*17/*17)

Model/estimate	η_CL/F_ (%CV)	OFV^a^	LLD^b^	*p* value
CL/F= θ_1_ (basic model)		−604.54	–	
θ_1_ =8.69	92.95			
CL/F = θ_1_ × θ_2_^G1^		−616.36	11.82	<0.001
θ_1_ = 7.51	110.91			
θ_2_ = 0.203				
CL/F = θ_1_ x θ_2_^G1^ x θ_3_^G2^		−621.44	5.08	<0.05
θ_1_ = 7.57	113.14			
θ_2_ = 0.263				
θ_3_ =1.70				
CL/F = θ_1_ x θ_2_^G1^ x θ_3_^G2^ x θ_4_^G3^		−627.39	5.95	<0.05
θ_1_ = 7.94	138.56			
θ_2_ = 0.205				
θ_3_ = 1.74				
θ_4_ = 2.00				
CL/F = θ_1_ × θ_2_^G1^ × θ_3_^G2^ × θ_4_^G3^ × θ_5_^G4^		−829.99	202.6	<0.001
θ_1_ = 17.1	31.05			
θ_2_ = 0.463				
θ_3_ = 0.974				
θ_4_ = 0.673				
θ_5_ = 0.196				
CL/F = θ_1_ × θ_2_^G1^ × θ_3_^G2^ × θ_4_^G3^ × θ_5_^G4^ × θ_6_^G5^		−864.97	34.98	<0.001
θ_1_ = 12.7	28.14			
θ_2_ = 0.59				
θ_3_ = 1.23				
θ_4_ =0.86				
θ_5_ = 0.25				
θ_6_ = 2.27				

^a^Objective function value

^b^Difference in the -2 log likelihood in relation to the model above

**Table 4:** Final population model estimates of pantoprazole

Parameter	Estimate	%RSE
CL/F, L/h	12.5	8.88^a^
V_c_/F, L	8.66	35.80
k_a_ , h^-1^	1.09	28.44
t_lag ,_ h	1.45	0.99
Q/F, L/h	3.34	15.12
V_p_/F, L	18.3	27.05
Interindividual variability (%CV)		
η_CL/F_	35.92	24.69^b^
η_V/F_	112.25	12.62
η_ka_	6.03	70.94
η_tlag_	1.92	36.14
Residual variability		
σ_1_ (%CV)	40.13	
σ_2_ (mg/L)	0.07	

^a^Calculated as 100%xSE/estimate

^b^Calculated as 100%xSE(omega)/(2*estimate(omega)

**Fig. 2:**
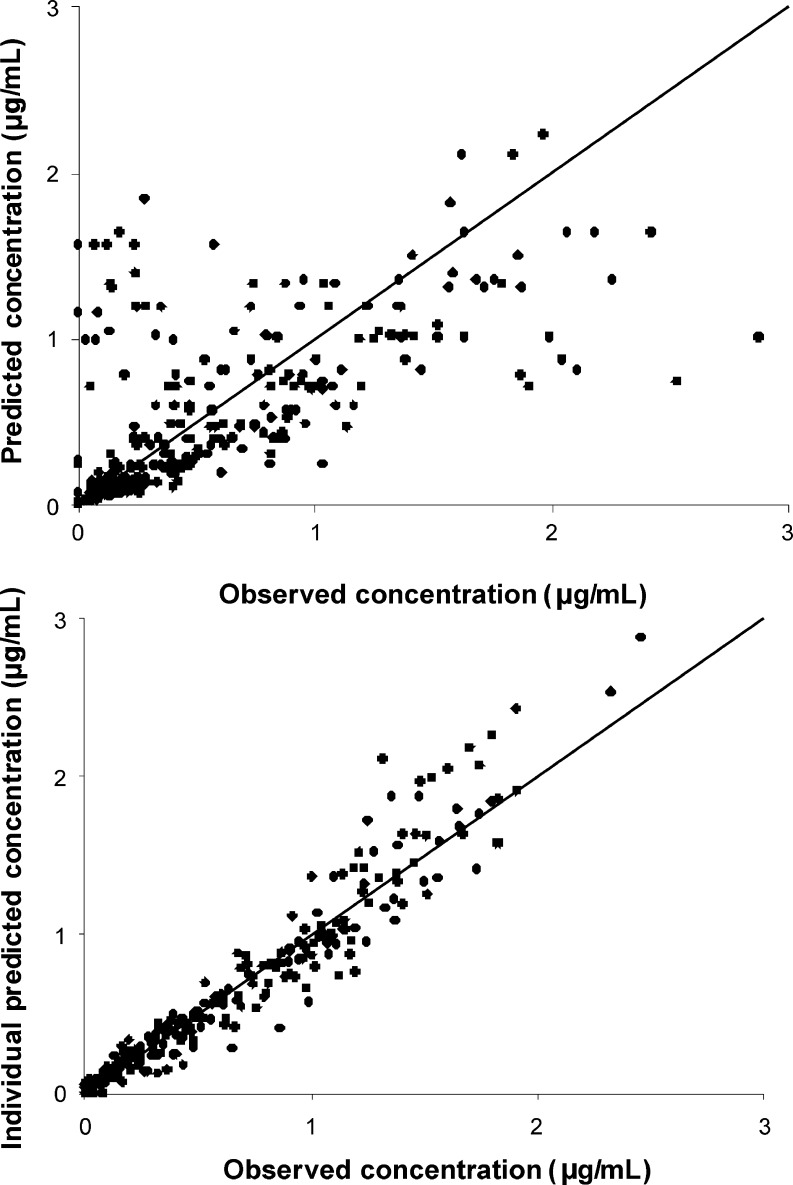
Population (*upper panel*) and individual (*lower panel*) models predicted pantoprazole concentrations versus observed concentrations. The *solid line* represents the line of unity

**Fig. 3:**
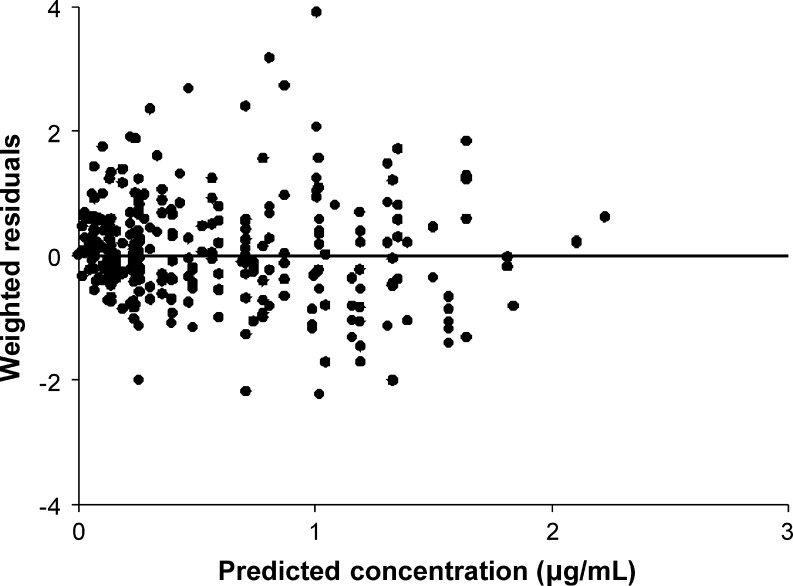
Weighted residuals versus population model predicted pantoprazole concentrations

Figures 2 and 3 represent the relationship between the population (PRED) and the individual (IPRED) model predicted pantoprazole concentrations versus observed concentrations and weighted residuals (WRES) as a function of PRED plots respectively. From Figs. 2 and 3, it seems that the final model where *CYP2C19* genotypes were included as covariates describes well concentration versus time profiles of pantoprazole. This can be confirmed both by the plot representing IPRED versus observed concentrations where the data points are aligned on the identity line (Fig. 2), and by the WRES versus the PRED relationship, which shows random scatter around zero (Fig. 3). Moreover, calculated epsilon shrinkage was low (12.6%), indicating that the diagnostic plot involving IPRED is relevant.

## Discussion

Definition of *CYP2C19* polymorphism is important for clinical practice as it was documented that the polymorphism is associated with the efficacy of the treatment of gastric acid-related disorders, with better clinical outcome in poor metabolizers, i.e., carriers of the CYP2C19*2 and CYP2C19*3 allele [14–17]. However, the pharmacokinetic studies did not involve subjects homozygous for the CYP2C19*17 allele, which seems to have a clinically significant impact, as it was proven for antiplatelet action of clopidogrel [18]. Sim et al. in the first report on the CYP2C19*17 allele in reporter vector experiments found an increased transcriptional activity of CYP2C19*17 allele in vivo in mice [4]. The clinical part of the study revealed up to 50–60% higher metabolic rates for omeprazole and mephenytoin in homozygotes for CYP2C19*17 allele than subjects homozygous for the CYP2C19*1 allele after a single dose of the drug.

Pantoprazole undergoes O-demethylation via CYP2C19, followed by sulfate conjugation and sulfone/sulfide formation [17]. Having in mind widespread clinical application of the drug it is very important to define the impact of *CYP2C19* polymorphism on its pharmacokinetics. The completed studies recruited healthy volunteers and patients being carriers of CYP2C19*1, CYP2C19*2, CYP2C19*3, and CYP2C19*17. However, the CYP2C19*17 allele was solely evaluated in a heterozygous setting. The study of Sim et al. [4], as well as our preliminary report [10], suggest that the CYP2C19*17/*17 state?? might significantly affect PPI pharmacokinetics. Sim et al. [4] demonstrated a higher omeprazole/5-hydroxyomeprazole metabolic ratio and Gawrońska-Szklarz et al. [10] higher blood concentrations of pantoprazole attained 3 h after oral drug administration in CYP2C19*17/*17 subjects in comparison to wild-type CYP2C19*1/*1 homozygotes. Our former study [10] evaluated pantoprazole concentration at a single time point as stated above, giving only preliminary information on *CYP2C19* polymorphism effects on the drug concentrations in patients stratified according to genotype (however, CYP2C19*2/*2 patients were not available for drug concentration studies). The present study analyzes pharmacokinetic data that enable better definition of the role of the *CYP2C19* polymorphism in pantoprazole pharmacokinetics, and also involves CYP2C19*2/*2 volunteers. It is demonstrated that polymorphism of *CYP2C19* affects pharmacokinetics of pantoprazole. The impact was documented for CYP2C19*1/*2, CYP2C19*2/*2, and CYP2C19*17/*17 genotypes; however, statistical analysis did not reveal any prominent effects of CYP2C19*1/17* and CYP2C19*2/*17 genotypes. Carriers of the genotype CYP2C19*2/*2 were characterized by higher drug concentrations beginning from 3.5 h after oral pantoprazole administration, and an increase in AUC and MRT were seen in CYP2C19*1/*2 and CYP2C19*2/*2 subjects compared with *CYP2C19* wild-type individuals. Also, a significantly higher C_max_ was observed in CYP2C19*1/*2 volunteers. These findings are in line with the reports of Hunfeld et al. [7, 8], who documented higher, although not significantly different AUC, in CYP2C19*1/*2 patients in 2008 [7], and significant differences between CYP2C19*1/*2 and CYP2C19*1/*1 in 2010 [8]. Similar findings were reported by Kearns et al. [9] from the pediatric population, where AUC values for pantoprazole were significantly elevated in CYP2C19*1/*2 children. Results from homozygous CYP2C19*2 patients were also reported in the aforementioned studies, i.e. Hunfeld et al. [7] found a higher AUC of pantoprazole in those subjects, although the differences did not reach statistical significance. Our observations do confirm this finding, but at a statistically significant level, and the results are in keeping with pediatric observations by Kearns et al. [9].

According to the results of the statistical analysis performed in the present study heterozygosity for the CYP2C19*17 allele does not affect the pharmacokinetics of pantoprazole. Both subjects with CYP2C19*1/*17 and CYP2C19*2/*17 were characterized with similar concentration–time curves and values of the calculated pharmacokinetic parameters as CYP2C19*1/*1 carriers. These observations are in line with the report by Hunfeld et al. [7], who did not find an effect of CYP2C19*1/*17 on the AUC of the drug, and by Kearns et al. [9] for CYP2C19*1/*17 and CYP2C19*2/*17 genotypes and AUC of pantoprazole. Based on these reports and our own results it can be concluded that heterozygosity for the CYP2C19*17 allele do not affect considerably pantoprazole kinetics.

Our population modeling confirmed the influence of the *1/*2, *2/*2, and *17/*17 genotypes on the pharmacokinetics of pantoprazole. Based on the changes in objective function value, a strong impact on pantoprazole oral clearance was observed in both homozygotes *2/*2 and *17/*17, whereas in genotype *1/*2 the effect was significant, but less evident.

Our study is the first one evaluating an effect of CYP2C19*17/*17 on pantoprazole plasma levels. It is revealed that the drug concentrations in carriers of the CYP2C19*17/*17 genotype are significantly higher than in the *CYP2C19* wild-type genotype beginning from 3 h after single oral administration of 40 mg of the drug. The calculated pharmacokinetic parameters also demonstrate lower drug accumulation and accelerated elimination in CYP2C19*17/*17 vs. CYP2C19*1/*1 subjects. An effect of CYP2C19*17 polymorphism was also evaluated for another PPIs, i.e., omeprazole. In CYP2C19*17/*17 subjects, it was found to influence the metabolic ratio of omeprazole/5-hydroxyomeprazole [4] and had no effects on esomeprazole [19] and omeprazole [20] pharmacokinetics, whereas for CYP2C19*17 heterozygosity no impact on omeprazole pharmacokinetics was documented [7, 9].

Like the CYP2C19*17/*17 genotype, a prominent effect of the CYP2C19*2/*2 genotype was observed in the present study. Homozygous carriers of the CYP2C19*2 allele were characterized by higher plasma levels of pantoprazole along with a decreased λ_z_, prolongation of MRT and t_½_, and a rise in the AUC in comparison to the CYP2C19*1/*1 genotype. Effects of poor *CYP2C19* status on the pharmacokinetics of other PPIs were also documented for omeprazole [7, 19, 20], esomeprazole [8], rabeprazole [21], and lansoprazole [7, 21, 22].

These observations indicate that the *CYP2C19* status affects the pharmacokinetics of pantoprazole. Moreover, our findings document that 57% of the intersubject variability in pantoprazole clearance can be explained by the *CYP2C19* genotype status. The results also support clinical observations indicating the variability of cure rates of gastric acid-related disorders treated with pantoprazole. It was found that the CYP2C19*2/*2 genotype was associated with higher *Helicobacter pylori* eradication rates, whereas no associations were noted for CYP2C19*1/*17 and CYP2C19*17/*17 patients [23]. Our previous study also indicated an influence of the *CYP2C19* polymorphism on *Helicobacter pylori* eradication, since carriers of *1/*1, *1/*17, and *17/*17 were at a higher risk of treatment failure in comparison to *2/*2, *1/*2, and *2/*17 patients [10].

The results of the present study suggest that *CYP2C19* genotyping should be used as a predictor of pantoprazole concentrations, and thus it may be useful as a dose adjustment tool. However, it should be stated that the results of the present study refer to single-dose pantoprazole pharmacokinetics, and an effect of *CYP2C19* polymorphism on multiple dosing should be addressed in further investigations.
